# Supplementary material for: Data-driven discovery of the spatial scales of habitat choice by elephants
Source: PeerJ. 2014 Aug 19;2:e504. doi: 10.7717/peerj.504 (PMC4145068; doi:10.7717/peerj.504)
Supplement: Supplemental Information 1 [file peerj-02-504-s001.pdf]

The following functions calculate the log-likelihood of a movement (or list of movements) using the conditional logistic model.

The function ‘logLikelihoodOneMovement’ takes as input (1) a list of data points for one or more variables that represent (a) the chosen location, indicated with a ‘1’ at the end of the list of values, and (b) a random subset of not-chosen locations within the local neighborhood, indicated with a ‘0’ at the end of each list of values (xd), and (2) a list of unknown parameters (B1, B2...) to be estimated (betas), which correspond to the number of variables.

```
logLikelihoodOneMovement[xd_, betas_] := Module[{numerator, logP},
  numerator = xd[[All, 1 ;; -2]].betas;
  logP = Chop[Pick[numerator, xd[[All, -1]], 1][[1]] - Log[Total[Exp[numerator]]]]]
```

The function ‘logLikelihoodAllMovements’ simply sums the log-likelihood values for each movement in the list of xd values (localData from below).

```
logLikelihoodAllMovements[xd_, betas_] :=
 Total[Map[logLikelihoodOneMovement[#, betas] &, xd]]
```

The smoothing function ‘landscapeSmooth’ takes as input a raster layer of environmental landscape data, the size of the radius (r), the decay parameter (f), and a binary array representing valid pixel locations within the landscape (validArray). The output is a smoothed version of the landscape.

```
landscapeSmooth[landscape_, r_, f_, validArray_] := Module[
  {landscapeImage, rInt, smooth, landscapeSmoothImage, landscapeSmooth, numPixels},

  (* The landscape is converted into an image. *)
  landscapeImage = Image[landscape];

  (* The radius must be an integer. *)
  rInt = Round[r];

  (* Convert the rxr square smoothing kernel into
   a circle by assigning a value of 0 to pixels that have a
   distance from the center pixel that is greater than the radius;
  for the remaining pixels, multiply their distance from the
   center pixel by the decay parameter divided by the radius,
  and subtract from 1 to determine how much weight each pixel contributes
    to the average. For f = 0, all pixels will have a value of 1. *)
  smooth = N[Table[If[EuclideanDistance[{i, j}, {rInt + 1, rInt + 1}] > rInt,
      0, (1 - EuclideanDistance[{i, j}, {rInt + 1, rInt + 1}] * f / rInt)],
     {i, 1, 2 * rInt + 1}, {j, 1, 2 * rInt + 1}]];

  (* Smooth the landscape image using 'smooth' as the kernel. *)
  landscapeSmoothImage = ImageConvolve[landscapeImage, smooth, Padding → 0.];

  (* Smooth the image of the valid array using 'smooth' as the kernel. *)
  numPixels = ImageConvolve[Image[validArray], smooth, Padding → 0.];

  (* Set pixels outside the valid array to 0; for valid pixels,
  divide the smoothed image data by the smoothed valid array data. *)
  landscapeSmooth = MapThread[If[#1 == 0, 0, If[#3 == 0, 0, #2 / #3]] &,
    {validArray, ImageData[landscapeSmoothImage], ImageData[numPixels]}, 2]]
```

The function ‘distanceFit’ optimizes the power value of the ‘distance from current location’ parameter. It takes as input a list of movement pairs, the radius of available neighbor pixel choices (here, 10), power

values over which to optimize distance (here, d ranges from 0.25 to 1 in steps of 0.05), the size of the random sample of neighbor pixels (here, 30), and the valid array. The output is each distance power (d) and the likelihood.

```
distanceFit[movements_, neighborRadius_, distanceVals_, sample_, validArray_] :=
 Module[{r, c, n, distancePowerLikelihood, rawResults, localData,
   startPoint, endPoint, distanceArray, localNeighborhood, randomPositions,
   randomNeighborhood, notPickedLocations, pickedLocation, logL},

  (* Determine the size of the landscape. *)
  {r, c} = Dimensions[validArray];

  (* Only one model (distance from current location) is being fit. *)
  n = 1;

  (* Loop over each of the distance powers (d). *)
  distancePowerLikelihood = Table[

    Print["Distance Power " <> ToString[d]];

    rawResults =

     (* Loop over movement data (i). *)
     {localData = ParallelTable[

        startPoint = movements[[i, 1]];
        endPoint = movements[[i, 2]];

        (* Create an array of the distances from the start point. *)
        distanceArray = ImageData[DistanceTransform[
           Image[ReplacePart[Table[1, {r}, {c}], startPoint → 0]]]];

        (* Find local neighborhood of
         start point by dilation (restricted to validArray.) *)
        localNeighborhood = Round[ImageData[ImageMultiply[
            Dilation[Image[ReplacePart[Table[0, {r}, {c}], startPoint → 1]],
             DiskMatrix[neighborRadius]], Image[validArray]]]];
        (* Eliminate end point (leaving only not-chosen locations). *)
        localNeighborhood = ReplacePart[localNeighborhood, endPoint → 0];

        (* Choose random subset of local neighborhood. *)
        randomPositions =
         RandomSample[Range[1, Length[Position[localNeighborhood, 1]]], sample];
        randomNeighborhood = ReplacePart[localNeighborhood,
          Position[localNeighborhood, 1][[randomPositions]] → 2];

        (* Pick distance from not-chosen locations. *)
        notPickedLocations =
         N[Flatten[Pick[distanceArray^d, randomNeighborhood, 2]]];
        (* Add zeros to the ends of the not-chosen data lines. *)
        notPickedLocations = Map[Append[{#}, 0] &, notPickedLocations];

        (* Pick distance from chosen location. *)
        pickedLocation = N[(distanceArray^d)[[endPoint[[1]], endPoint[[2]]]]];
        (* Add one to the end of the chosen data line. *)
```

```
        pickedLocation = Append[{pickedLocation}, 1];

        (* Put the chosen data line in front of the not-
         chosen data lines for that movement. *)
        Prepend[notPickedLocations, pickedLocation], {i, 1, Length[movements]}];

      (* Find the maximum log-likelihood of the parameter B1,
      given the local data from above. *)
      logL = Chop[logLikelihoodAllMovements[localData, {B1}]];
      FindMaximum[logL, {B1}, Method → "PrincipalAxis"]};

    Print[Flatten[rawResults, 1]];
    {d, rawResults[[1, 1]]}, {d, N[distanceVals]}]]
```

The function ‘smoothFit’ optimizes the smoothing radius for a single landscape variable. It takes as input a list of movement pairs, a raster layer of environmental landscape data, the radius of available neighbor choices, the smoothing radii over which to optimize (here, j ranges from 0 to 20 in steps of 1), the optimal distance power (from distanceFit), the size of the random sample of neighbor pixels, the valid array, and the label “Unknown” to treat pixels outside the boundaries of the landscape as unknown (instead of 0) when smoothing. The output is each radius (j) and the likelihood.

```
smoothFit[movements_, landscapes_, neighborRadius_,
  radiusScales_, distancePower_, sample_, validArray_, boundaries_] :=
 Module[{r, c, n, models, variableList, randomPositions, start, end,
   neighborhood, rawResults, smoothLandscapes, localData, startPoint, endPoint,
   distanceArray, localNeighborhood, randomNeighborhood, notPickedLocations,
   pickedLocation, allFits, logL, variableSpecification, out1},

  (* Determine the size of the landscape. *)
  {r, c} = Dimensions[validArray];

  (* The number of variables (including distance and squared term). *)
  n = 3;

  (* Models as binary codes. *)
  models = Partition[Flatten[
     Map[Prepend[#, 1] &, Sort[Tuples[{0, 1}, n - 1], Total[#1] < Total[#2] &]]], n];

  (* Lists of variable symbols
   {B1, B2, etc.} for each model (for FindMaximum input). *)
  variableList = ToExpression[Table[StringJoin["B", ToString[i]], {i, 1, n}]];

  (* Generate a list of the random positions to use when sampling
   neighbor pixels, looped over movement data (i). This ensures the
   same random pixels are sampled for each smoothing radius. *)
  randomPositions = Table[
    start = movements[[i, 1]];
    end = movements[[i, 2]];

    neighborhood = Round[ImageData[
       ImageMultiply[Dilation[Image[ReplacePart[Table[0, {r}, {c}], start → 1]],
         DiskMatrix[neighborRadius]], Image[validArray]]]];
    neighborhood = ReplacePart[neighborhood, end → 0];

    RandomSample[Range[1, Length[Position[neighborhood, 1]]], sample],
```

```
    {i, 1, Length[movements]}];

  (* Loop over smoothing radii (j). *)
  rawResults = Table[

    Print["Smoothing Radius " <> ToString[j]];

    Which[boundaries == "Unknown",
     smoothLandscapes =
      If[j > 0, landscapeSmooth[landscapes, j, 0., validArray], landscapes],
     boundaries == "Zero",
     smoothLandscapes = If[j > 0, landscapeSmooth[landscapes, j, 0.], landscapes]];

    (* Loop over movement data (i). *)
    localData = ParallelTable[

      startPoint = movements[[i, 1]];
      endPoint = movements[[i, 2]];

      (* Create an array of distances from the start point. *)
      distanceArray = ImageData[DistanceTransform[
         Image[ReplacePart[Table[1, {r}, {c}], startPoint → 0]]]];

      (* Find local neighborhood of
       start point by dilation (restricted to validArray). *)
      localNeighborhood = Round[ImageData[ImageMultiply[
          Dilation[Image[ReplacePart[Table[0, {r}, {c}], startPoint → 1]],
           DiskMatrix[neighborRadius]], Image[validArray]]]];
      (* Eliminate end point (leaving only not-chosen locations). *)
      localNeighborhood = ReplacePart[localNeighborhood, endPoint → 0];
      (* Choose random subset of local neighborhood. *)
      randomNeighborhood = ReplacePart[localNeighborhood,
        Position[localNeighborhood, 1][[randomPositions[[i]]]] → 2];

      (* Pick landscape data from not-chosen locations. *)
      notPickedLocations =
       N[Transpose[Map[Flatten[Pick[#, randomNeighborhood, 2]] &,
          Prepend[Append[{smoothLandscapes}, smoothLandscapes^2],
           distanceArray^distancePower]]]];
      (* Add zeros to the ends of the not-chosen data lines. *)
      notPickedLocations = Map[Append[#, 0] &, notPickedLocations];

      (* Pick landscape data from chosen location. *)
      pickedLocation = N[
        Map[#[[endPoint[[1]], endPoint[[2]]]] &, Prepend[Append[{smoothLandscapes},
           smoothLandscapes^2], distanceArray^distancePower]]];
      (* Add one to the ends of the chosen data line. *)
      pickedLocation = Append[pickedLocation, 1];
      (* Put the chosen data line in front of the not-
       chosen data lines for that movement. *)
      Prepend[notPickedLocations, pickedLocation], {i, 1, Length[movements]}];

    Print["Fitting"];

    (* Loop over all models (m). *)
```

```
    allFits = ParallelTable[
      (* Create symbolic representation of log-likelihood function. *)
      logL =
       Chop[logLikelihoodAllMovements[localData, variableList * models[[m]]]];
      (* Create variable specification for the model. *)
      variableSpecification = Pick[variableList, models[[m]], 1];
      (* Fit model. *)
      out1 = FindMaximum[logL, variableSpecification, Method → "PrincipalAxis"];
      out1, {m, 1, Length[models]}];

    allFits,

    {j, N[radiusScales]}];

  rawResults]
```

The function ‘decayFit’ optimizes the decay parameter for a single smoothing radius of a particular landscape variable. It takes as input a list of movement pairs, a raster layer of environmental landscape data, the radius of available neighbor pixel choices, an optimal smoothing radius (from smoothFit), the range of decay values over which to optimize (here, f ranges 0 to 1 in steps of 0.1), the optimal distance power (from distanceFit), the size of the random sample of neighbor pixels, the valid array, and the label “Unknown.” The output is each decay value (f) and the likelihood.

```
decayFit[movements_, landscapes_, neighborRadius_, radiusScale_,
  decayScales_, distancePower_, sample_, validArray_, boundaries_] :=
 Module[{r, c, n, models, variableList, randomPositions, start, end,
   neighborhood, rawResults, smoothLandscapes, localData, startPoint, endPoint,
   distanceArray, localNeighborhood, randomNeighborhood, notPickedLocations,
   pickedLocation, allFits, logL, variableSpecification, out1},

  (* Determine the size of landscape. *)
  {r, c} = Dimensions[validArray];

  (* The number of variables (including distance). *)
  n = 2;

  (* Models as binary codes. *)
  models = Partition[Flatten[
     Map[Prepend[#, 1] &, Sort[Tuples[{0, 1}, n - 1], Total[#1] < Total[#2] &]]], n];

  (* Lists of variable symbols
   {B1, B2, etc.} for each model (for FindMaximum input). *)
  variableList = ToExpression[Table[StringJoin["B", ToString[i]], {i, 1, n}]];

  (* Generate a list of the random positions to use when sampling
   neighbor pixels, looped over movement data (i). This ensures
   the same random pixels are sampled for each smoothing decay. *)
  randomPositions = Table[
    start = movements[[i, 1]];
    end = movements[[i, 2]];

    neighborhood = Round[ImageData[
       ImageMultiply[Dilation[Image[ReplacePart[Table[0, {r}, {c}], start → 1]],
         DiskMatrix[neighborRadius]], Image[validArray]]]];
    neighborhood = ReplacePart[neighborhood, end → 0];
```

```
    RandomSample[Range[1, Length[Position[neighborhood, 1]]], sample],
    {i, 1, Length[movements]}];

  (* Loop over decay (f). *)
  rawResults = Table[

    Print["Smoothing Decay " <> ToString[f]];

    (* Smooth the landscape at the given radius with the current value of f,
    using the "Unknown" method. *)
    Which[boundaries == "Unknown",
     smoothLandscapes = landscapeSmooth[landscapes, radiusScale, f, validArray],
     boundaries == "Zero",
     smoothLandscapes = landscapeSmooth[landscapes, radiusScale, f]];

    (* Loop over movement data (i). *)
    localData = ParallelTable[

      startPoint = movements[[i, 1]];
      endPoint = movements[[i, 2]];

      (* Create an array of the distances from the start point. *)
      distanceArray = ImageData[DistanceTransform[
         Image[ReplacePart[Table[1, {r}, {c}], startPoint → 0]]]];

      (* Find local neighborhood of
       start point by dilation (restricted to validArray). *)
      localNeighborhood = Round[ImageData[ImageMultiply[
          Dilation[Image[ReplacePart[Table[0, {r}, {c}], startPoint → 1]],
           DiskMatrix[10]], Image[validArray]]]];
      (* Eliminate end point (leaving only not-chosen locations). *)
      localNeighborhood = ReplacePart[localNeighborhood, endPoint → 0];
      (* Choose random subset of local neighborhood *)
      randomNeighborhood = ReplacePart[localNeighborhood,
        Position[localNeighborhood, 1][[randomPositions[[i]]]] → 2];

      (* Pick landscape data from not-chosen locations. *)
      notPickedLocations =
       N[Transpose[Map[Flatten[Pick[#, randomNeighborhood, 2]] &,
          Prepend[{smoothLandscapes}, distanceArray^distancePower]]]];
      (* Add zeros to the ends of the not-chosen data lines. *)
      notPickedLocations = Map[Append[#, 0] &, notPickedLocations];

      (* Pick landscape data from chosen location. *)
      pickedLocation = N[Map[#[[endPoint[[1]], endPoint[[2]]]] &,
         Prepend[{smoothLandscapes}, distanceArray^distancePower]]];
      (* Add one to the ends of the chosen data line. *)
      pickedLocation = Append[pickedLocation, 1];
      (* Put the chosen data line in front of the not-
       chosen data lines for that movement. *)
      Prepend[notPickedLocations, pickedLocation], {i, 1, Length[movements]}];

    Print["Fitting"];
```

```
    (* Loop over all models (m). *)
    allFits = ParallelTable[
      (* Create symbolic representation of log-likelihood function. *)
      logL =
       Chop[logLikelihoodAllMovements[localData, variableList * models[[m]]]];
      (* Create variable specifiation for the model. *)
      variableSpecification = If[f == Min[N[decayScales]], Pick[variableList,
         models[[m]], 1], Transpose[{Pick[variableList, models[[m]], 1],
          Pick[variableList, models[[m]], 1] /. allFits[[m, 2]]}]];
      (* Fit model. *)
      out1 = FindMaximum[logL, variableSpecification, Method → "PrincipalAxis"];
      out1, {m, 1, Length[models]}];

    allFits,

    {f, N[decayScales]}];

  rawResults]
```

The function ‘modelFit’ fits each combination of landscape variables. It takes as input a list of movement pairs, a list of raster layers for each environmental landscape variable at its optimal scale(s) and decay, the radius of available neighbor pixel choices, the optimal distance power (from distanceFit), the size of the random sample of neighbor pixels, and the valid array. The output is a list of likelihood values for each model.

```
modelFit[movements_, landscapes_,
  neighborRadius_, distancePower_, sample_, validArray_] :=
 Module[{r, c, n, models, variableList, randomPositions, start, end, neighborhood,
   localData, startPoint, endPoint, distanceArray,
   localNeighborhood, randomNeighborhood, notPickedLocations,
   pickedLocation, allFits, logL, variableSpecification, out1},

  (* Determine the size of landscape. *)
  {r, c} = Dimensions[validArray];

  (* The number of variables (including distance). *)
  n = Length[landscapes] + 1;

  (* Models as binary codes. *)
  models = Partition[Flatten[
     Map[Prepend[#, 1] &, Sort[Tuples[{0, 1}, n - 1], Total[#1] < Total[#2] &]]], n];

  (* List of variable symbols
   {B1, B2, etc.} for each model (for FindMaximum input). *)
  variableList = ToExpression[Table[StringJoin["B", ToString[i]], {i, 1, n}]];

  (* Loop over movement data (i). *)
  localData = ParallelTable[

    startPoint = movements[[i, 1]];
    endPoint = movements[[i, 2]];

    (* Create an array of the distances from the start point. *)
    distanceArray = ImageData[
      DistanceTransform[Image[ReplacePart[Table[1, {r}, {c}], startPoint → 0]]]];
```

```
(* Find local neighborhood of
 start point by dilation (restricted to validArray). *)
localNeighborhood = Round[ImageData[ImageMultiply[
    Dilation[Image[ReplacePart[Table[0, {r}, {c}], startPoint → 1]],
     DiskMatrix[neighborRadius]], Image[validArray]]]];
(* Eliminate end point (leaving only not-chosen locations). *)
localNeighborhood = ReplacePart[localNeighborhood, endPoint → 0];

(* Choose random subset of local neighborhood. *)
randomPositions =
 RandomSample[Range[1, Length[Position[localNeighborhood, 1]]], sample];
randomNeighborhood = ReplacePart[localNeighborhood,
  Position[localNeighborhood, 1][[randomPositions]] → 2];

(* Pick landscape data from not-chosen locations. *)
notPickedLocations = N[Transpose[Map[Flatten[Pick[#, randomNeighborhood, 2]] &,
    Prepend[landscapes, distanceArray^distancePower]]]];
(* Add zeros to the ends of the not-chosen data lines. *)
notPickedLocations = Map[Append[#, 0] &, notPickedLocations];

(* Pick landscape data from chosen location. *)
pickedLocation = N[Map[#[[endPoint[[1]], endPoint[[2]]]] &,
   Prepend[landscapes, distanceArray^distancePower]]];
(* Add one to the ends of the chosen data line. *)
pickedLocation = Append[pickedLocation, 1];

(* Put the chosen data line in front of the not-
 chosen data lines for that movement. *)
Prepend[notPickedLocations, pickedLocation], {i, 1, Length[movements]}];

(* Loop over all models (m). *)
allFits = ParallelTable[
  (* Create symbolic representation of log-likelihood function. *)
  logL = Chop[logLikelihoodAllMovements[localData, variableList * models[[m]]]];
  (* Create variable specifiation for the model. *)
  variableSpecification = Pick[variableList, models[[m]], 1];
  (* Fit model. *)
  out1 = FindMaximum[logL, variableSpecification, Method → "PrincipalAxis"];
  out1, {m, 1, Length[models]}];

allFits
]
```

The function ‘modelAIC’ takes as input the list of likelihood values for each model (from modelFit) and a list of variable names. The output is a table displaying the AIC score of the best model and the parameter values of each variable that is in that model.

```
modelAIC[results_, variableNames_] :=
 Module[{n, models, variableList, penalties, resultsAIC, bestModel},

  n = Length[variableNames] + 1;
  models = Partition[Flatten[
     Map[Prepend[#, 1] &, Sort[Tuples[{0, 1}, n - 1], Total[#1] < Total[#2] &]]], n];
  variableList = ToExpression[Table[StringJoin["B", ToString[i]], {i, 1, n}]];
  penalties = 2 * Map[Total, models];
  resultsAIC = MapThread[Prepend[#1, -2 * #1[[1]] + #2] &, {results, penalties}];
  bestModel = Select[resultsAIC, #[[1]] == Min[resultsAIC[[All, 1]]] &];
  Style[TableForm[Map[If[NumericQ[#], Round[#, 0.01], #] &,
     Map[Join[{#1[[1]]}, variableList /. #1[[3]]] &, bestModel], {2}],
    TableHeadings → {None, Join[{"AIC", "Distance"}, variableNames]},
    TableSpacing → {1.3, 2}], FontFamily → "Helvetica"]]
```

Alternatively, you can specify how many of the top models to display in the table.

```
modelAIC[results_, variableNames_, numberModels_] :=
 Module[{n, models, variableList, penalties, resultsAIC},

  n = Length[variableNames] + 1;
  models = Partition[Flatten[
     Map[Prepend[#, 1] &, Sort[Tuples[{0, 1}, n - 1], Total[#1] < Total[#2] &]]], n];
  variableList = ToExpression[Table[StringJoin["B", ToString[i]], {i, 1, n}]];
  penalties = 2 * Map[Total, models];
  resultsAIC = MapThread[Prepend[#1, -2 * #1[[1]] + #2] &, {results, penalties}];
  Style[TableForm[Map[If[NumericQ[#], Round[#, 0.01], #] &,
     Sort[Map[Join[{#1[[1]]}, variableList /. #1[[3]]] &, resultsAIC]][[
      1 ;; numberModels]], {2}],
    TableHeadings → {None, Join[{"AIC", "Distance"}, variableNames]},
    TableSpacing → {1.3, 2}], FontFamily → "Helvetica"]]
```

The function ‘parameterStatistics’ takes as input the list of likelihood values for each model (from modelFit) and a list of variable names. The output is a list of importance values for each variable and a list of the parameter-averaged value for each variable.

```
parameterStatistics[results_, variableNames_] :=
 Module[
  {n, models, variableList, logLList, aicList, minAIC, deltaAIC, expList, weightList,
   weightMatrix, importanceList, parameterMatrix, parameterAverageList},

  n = Length[variableNames] + 1;
  models = Partition[Flatten[
     Map[Prepend[#, 1] &, Sort[Tuples[{0, 1}, n - 1], Total[#1] < Total[#2] &]]], n];
  variableList = ToExpression[Table[StringJoin["B", ToString[i]], {i, 1, n}]];

  logLList = results[[All, 1]];
  aicList = MapThread[-2 * #1 + 2 * Total[#2] &, {logLList, models}];
  minAIC = Min[aicList];
  deltaAIC = aicList - minAIC;
  expList = Exp[-0.5 * deltaAIC];
  weightList = expList / Total[expList];
  weightMatrix = Map[weightList * # &, Transpose[models]];
  importanceList = Map[Total, weightMatrix];
  parameterMatrix = MapThread[(#1 * variableList) /. #2[[2]] &, {models, results}];
  parameterAverageList = MapThread[
    Total[#1 * #2] / Total[#2] &, {Transpose[parameterMatrix], weightMatrix}];
  Transpose[{importanceList, parameterAverageList}]]
```

The following code generates the local relative quality prediction maps. First, a mask surrounding the locations of individuals is created. It takes as input a list of movement pairs and the valid array.

```
landscapeMask[movements_, validArray_] :=
 Module[{positions, mask},

  positions =
   ReplacePart[Map[If[# == 0, 0, 0] &, validArray, {2}], movements[[All, 1]] → 1];
  mask = ImageMultiply[FillingTransform[
     Dilation[Image[positions], DiskMatrix[10]]], Image[validArray]];
  Round[ImageData[mask]]]
```

Next, the quality landscape is created by multiplying the landscape raster layers by the beta values (parameter estimates from modelFit).

```
qualityLandscape = Apply[Times, MapThread[Exp[#1 * #2] &, {landscapes, betas}]];
```

A local convolution of the quality landscape is created, and then the quality landscape is divided by the local convolution to get relative values.

```
qualityLandscapeLocal = ListConvolve[DiskMatrix[10], qualityLandscape, {11, 11}, 0];
qualityLandscape =
  MapThread[If[#2 == 0, 0, #1 / #2] &, {qualityLandscape, qualityLandscapeLocal}, 2];
```

The new quality landscape is multiplied by the location mask.

```
qualityLandscape = qualityLandscape * mask;
```

Scale the quality landscape to values between 0 and 1. Optional: Depending on the range of values, it may be appropriate to scale the quality landscape so that extremely large or small values do not bias the color distribution. Here we scale the quality landscape using 95% of the range of values, ignoring the largest and smallest 2.5%.

```
q1 = Quantile[Flatten[Pick[qualityLandscape, mask, 1]], 0.025];
q2 = Quantile[Flatten[Pick[qualityLandscape, mask, 1]], 0.975];
qualityLandscape = (qualityLandscape - q1) / (q2 - q1);
```

The map is generated using the dark rainbow color scheme based on the value of each pixel.

```
qualityMap = Image[Reverse[Map[Which[# ≤ 0, {0, 0, 0, 0},
       0 < # ≤ 0.1, {0.237736`, 0.340215`, 0.575113`, 1},
       0.1 < # ≤ 0.2, {0.253651`, 0.344893`, 0.558151`, 1},
       0.2 < # ≤ 0.3, {0.264425`, 0.423024`, 0.3849`, 1},
       0.3 < # ≤ 0.4, {0.291469`, 0.47717`, 0.271411`, 1},
       0.4 < # ≤ 0.5, {0.416394`, 0.555345`, 0.24182`, 1},
       0.5 < # ≤ 0.6, {0.624866`, 0.673302`, 0.264296`, 1},
       0.6 < # ≤ 0.7, {0.813033`, 0.766292`, 0.303458`, 1},
       0.7 < # ≤ 0.8, {0.877875`, 0.731045`, 0.326896`, 1},
       0.8 < # ≤ 0.9, {0.812807`, 0.518694`, 0.303459`, 1},
       # > 0.9, {0.72987`, 0.239399`, 0.230961`, 1}] &, qualityLandscape, {2}]],
   ColorSpace → "RGB"];
```

Finally, the map is shown with a gray-scale background of the landscape and is overlaid with the location end-points.

```
Show[background, qualityMap,
 ListPlot[Map[Reverse[#] - {0.5, 0.5} &, movements[[All, 2]]],
  PlotStyle → {PointSize[0.0035], Black}], ImageSize → 250]
```

The function ‘meanQuality’ generates the mean difference values for each pixel from the local relative quality map (to be used in the histograms). It takes as input a list of movement pairs, a list of raster layers for each environmental landscape variable at its optimal scale(s) and decay, a list of beta values for each landscape variable, and the valid array.

```
meanQuality[movements_, landscapes_, betas_, validArray_] :=
 Module[{start, end, r, c, localNeighborhood,
   localLandscape, localProb, selected, notSelected},

  (* Loop over movement data (i). *)
  Table[
   start = movements[[i, 1]];
   end = movements[[i, 2]];

   (* Determine the size of the landscape. *)
   {r, c} = Dimensions[validArray];

   (* Find local neighborhood of
    start point by dilation (restricted to validArray). *)
   localNeighborhood = Round[ImageData[ImageMultiply[Dilation[Image[ReplacePart[
         Table[0, {r}, {c}], start → 1]], DiskMatrix[10]], Image[validArray]]]];
   (* Mark end-point. *)
   localNeighborhood = ReplacePart[localNeighborhood, end → 2];

   (* Generate local landscapes based on the start and end locations. *)
   localLandscape = Map[Extract[#, Flatten[Append[{Position[localNeighborhood, 2]},
        Position[localNeighborhood, 1]], 1]] &, landscapes];

   (* Determine probability values of local landscape, scaled from 0-1. *)
   localProb = Apply[Times,
     MapThread[Exp[#1 * #2] / Total[Exp[#1 * #2]] &, {localLandscape, betas}]];
   localProb = (localProb - Min[localProb]) / (Max[localProb] - Min[localProb]);

   (* Identify selected and non-
    selected locations and determine mean difference. *)
   selected = localProb[[1]];
   notSelected = Rest[localProb];

   Mean[selected - notSelected], {i, 1, Length[movements]}]]
```

The following code generates the landscape-wide relative quality prediction maps. First, the quality landscape is created by multiplying the landscape raster layers by the beta values and summing them.

```
qualityLandscape = Total[MapThread[#1 * #2 &, {landscapes, betas}]];
```

Scale the quality landscape to values between 0 and 1. Optional: Depending on the range of values, it may be appropriate to scale the quality landscape so that extremely large or small values do not bias the color distribution. Here, we first determine the largest and smallest 2.5% of the data.

```
q1 = Quantile[Flatten[Pick[qualityLandscape, mask, 1]], 0.025];
q2 = Quantile[Flatten[Pick[qualityLandscape, mask, 1]], 0.975];
```

The landscape is then scaled from 0-1, with the smallest 2.5% all going to 0 and the largest 2.5% all going to 1.

```
qualityLandscape = Map[Which[# < q1, 0,
     # > q2, 1,
     q1 ≤ # ≤ q2, (# - q1) / (q2 - q1)] &, qualityLandscape, {2}];
```

The map is generated using the dark rainbow color scheme based on the value of each pixel.

```
qualityMap = ImageMultiply[
   Image[Map[Apply[List, ColorData["DarkRainbow"][#]] &, qualityLandscape, {2}]],
   Image[validArray]];
```

Finally, the map is overlaid with the location end-points.

```
Show[ImageReflect[qualityMap],
 ListPlot[Map[Reverse, movements[[All, 2]]], PlotStyle → {PointSize[0.003], White}]]
```
