# Supplementary material for: Data-driven discovery of the spatial scales of habitat choice by elephants
Source: PeerJ. 2014 Aug 19;2:e504. doi: 10.7717/peerj.504 (PMC4145068; doi:10.7717/peerj.504)
Supplement: Table S2 [file peerj-02-504-s002.pdf]

Maputo Females, Dry Season

Variable	Best model parameter value	Importance	Parameter–averaged value
Distance	–1.11	1.00	–1.11
Reeds	–0.07	0.43	–0.05
$Reeds^2$	-	0.42	–0.01
Trees	0.37	1.00	0.38
$Trees^2$	–0.39	1.00	–0.40
Water Distance	–0.29	0.64	–0.26
Water $Distance^2$	-	0.32	0.13
Settlement Distance	0.15	0.76	0.15

Maputo Females, Wet Season

Variable	Best model parameter value	Importance	Parameter–averaged value
Distance	–0.97	1.00	–0.97
Reeds	-	0.32	0.03
$Reeds^2$	-	0.31	0.01
Trees	0.31	0.99	0.33
$Trees^2$	–0.27	1.00	–0.28
Water Distance	–0.33	0.91	–0.31
Water $Distance^2$	-	0.27	0.01
Settlement Distance	-	0.34	0.06

Maputo Males, Dry Season

Variable	Best model parameter value	Importance	Parameter–averaged value
Distance	–1.06	1.00	–1.05
Reeds	-	0.31	0.01
$Reeds^2$	-	0.36	–0.02
Trees	0.36	1.00	0.33
$Trees^2$	–0.07	0.86	–0.06
Water Distance	-	0.33	0.05
Water $Distance^2$	–0.10	0.91	–0.1
Settlement Distance	-	0.32	0.05

Maputo Males, Wet Season

Variable	Best model parameter value	Importance	Parameter–averaged value
Distance	–1.06	1.00	–1.05
Reeds	-	0.42	0.05
$Reeds^2$	–0.05	0.81	–0.06
Trees	0.15	0.99	0.17
$Trees^2$	-	0.40	–0.02
Water Distance	-	0.38	–0.07
Water $Distance^2$	-	0.51	0.04
Settlement Distance	-	0.39	0.07

Etosha Females, Dry Season

Variable	Best model parameter value	Importance	Parameter–averaged value
Distance	–1.15	1.00	–1.15
Mopane	0.47	1.00	0.48
$Mopane^2$	-	0.41	0.06
A. nebrownii	0.32	1.00	0.32
A. nebrownii ^{2}	–0.03	0.95	–0.03
Acacia	0.17	0.98	0.23
Acacia^2	-	0.52	–0.03
Water Distance	–0.21	0.58	–0.25
Water $Distance^2$	0.16	0.84	0.19

Etosha Females, Wet Season

Variable	Best model parameter value	Importance	Parameter–averaged value
Distance	–1.40	1.00	–1.40
Mopane	0.15	0.88	0.14
$Mopane^2$	–0.13	0.91	–0.14
A. nebrownii	0.25	0.84	0.21
A. nebrownii ^{2}	–0.05	0.70	–0.05
Acacia	0.14	0.53	0.17
Acacia^2	-	0.33	–0.02
Water Distance	-	0.27	–0.03
Water $Distance^2$	-	0.37	–0.04
